# Supplementary material for: Thalamus orchestrates local acetylcholine-dependent dopamine release in the learning striatum
Source: bioRxiv. 2026 May 23:2026.05.08.723861. Originally published 2026 May 8. Preprint. [Version 2] doi: 10.64898/2026.05.08.723861 (PMC13174615; doi:10.64898/2026.05.08.723861)

Supplementary Figure 1

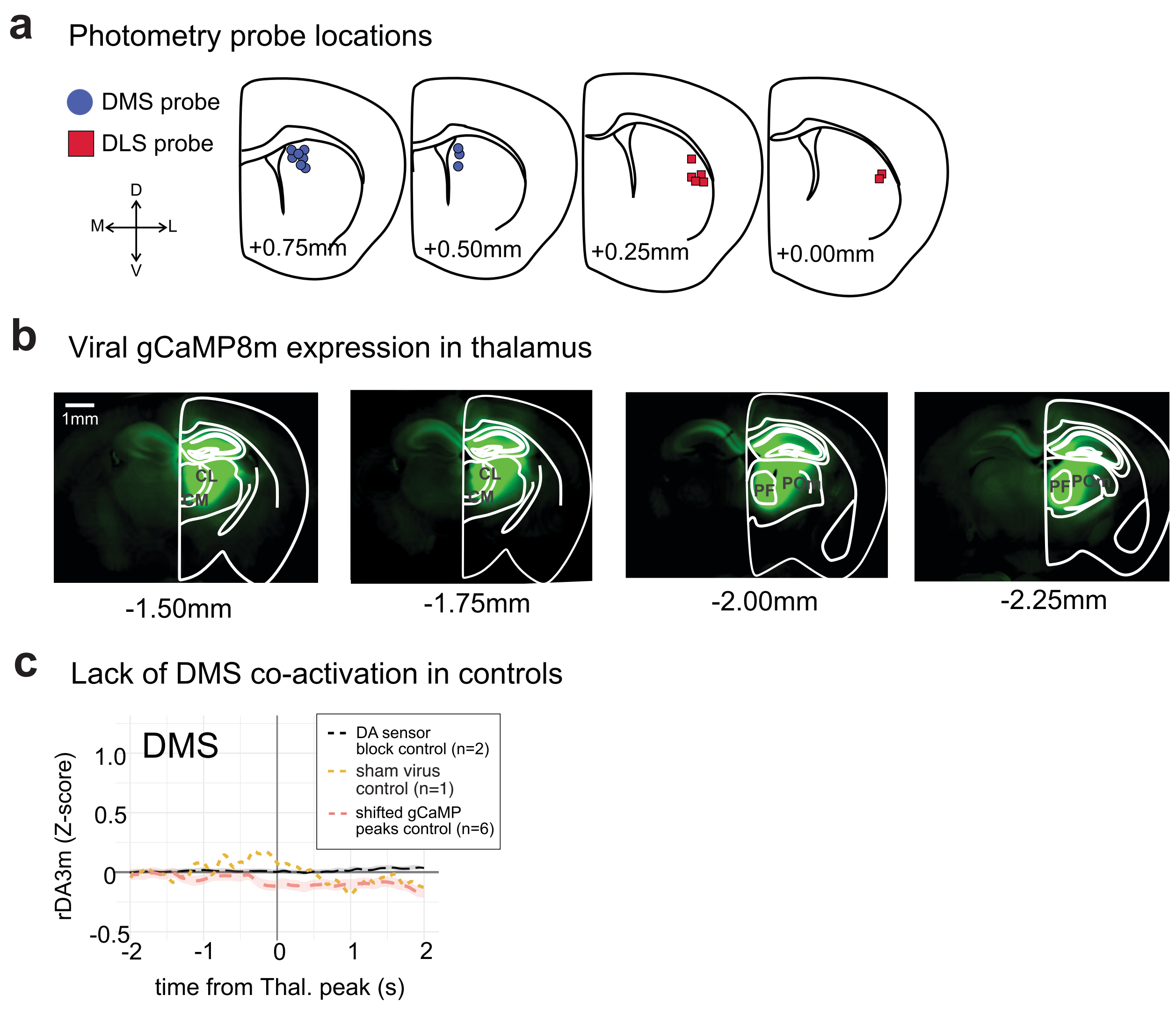

Supplementary Figure 2

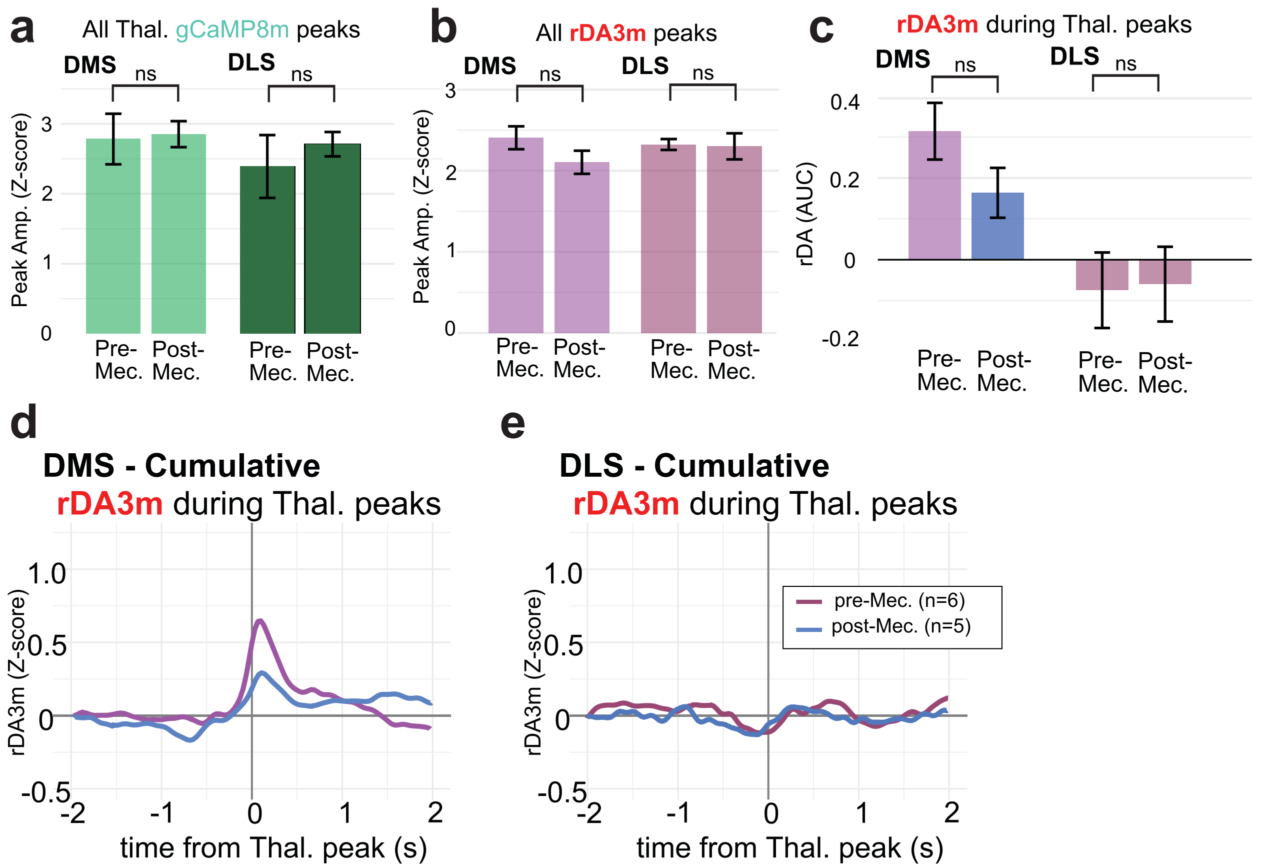

Supplementary Figure 3

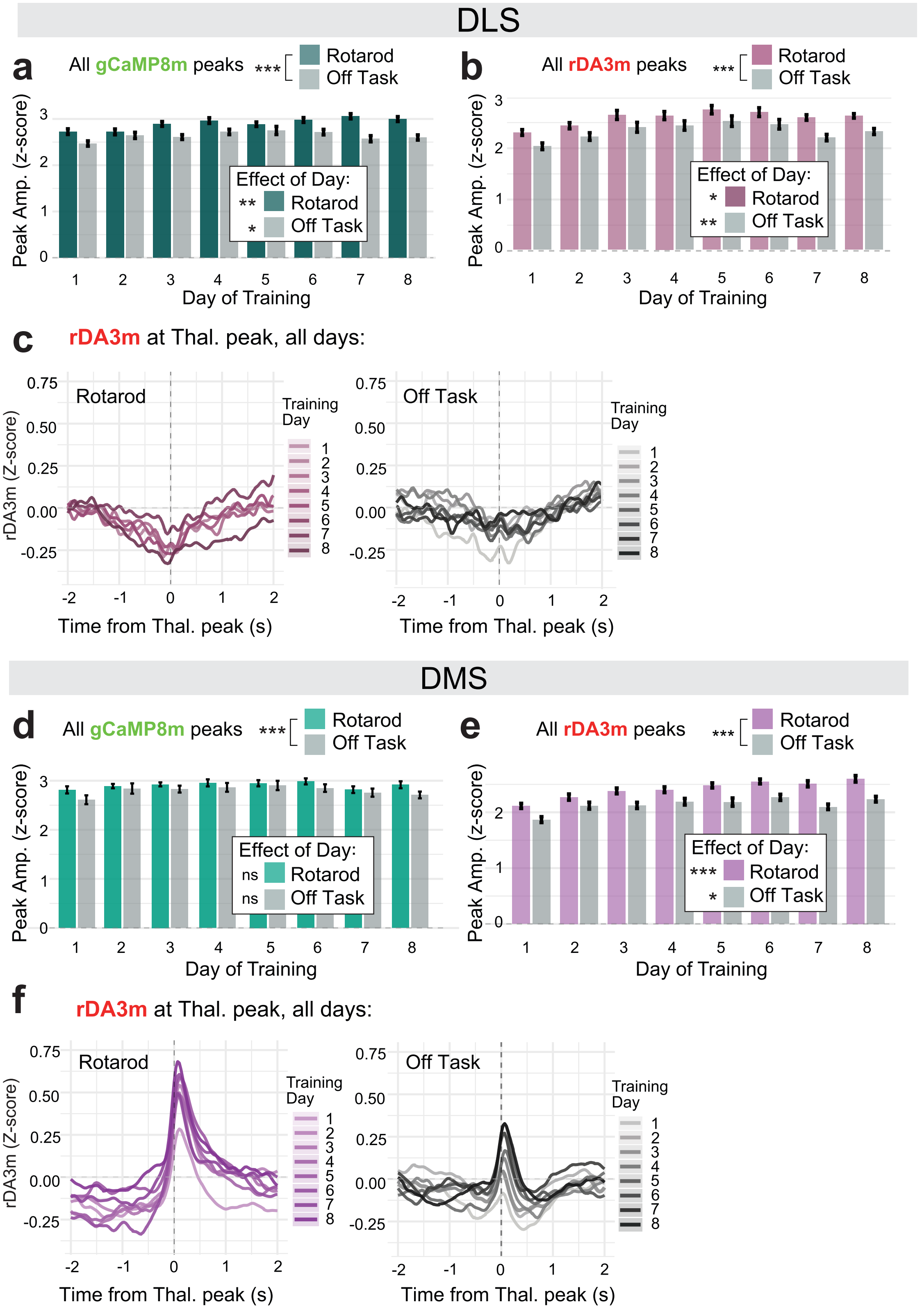

Supplementary Figure 4

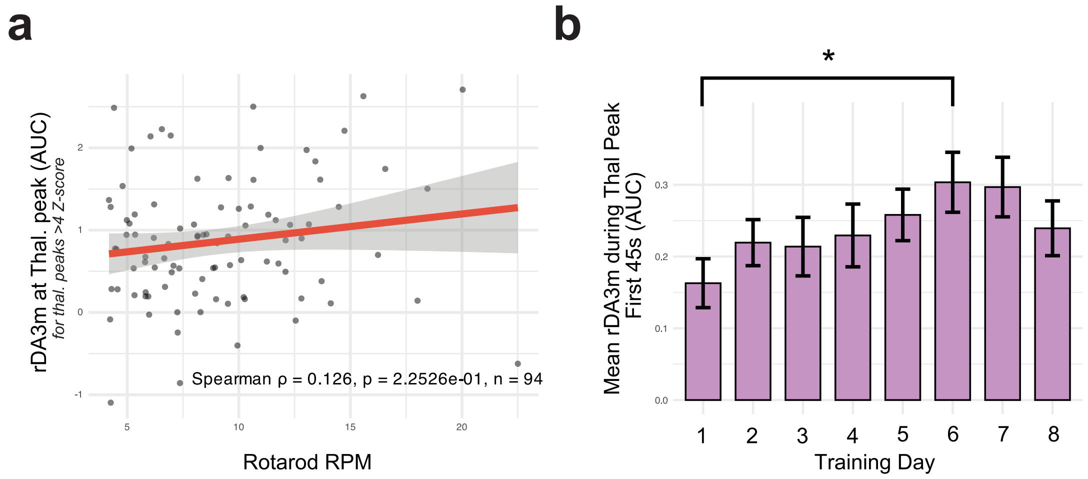

Supplementary Figure 5

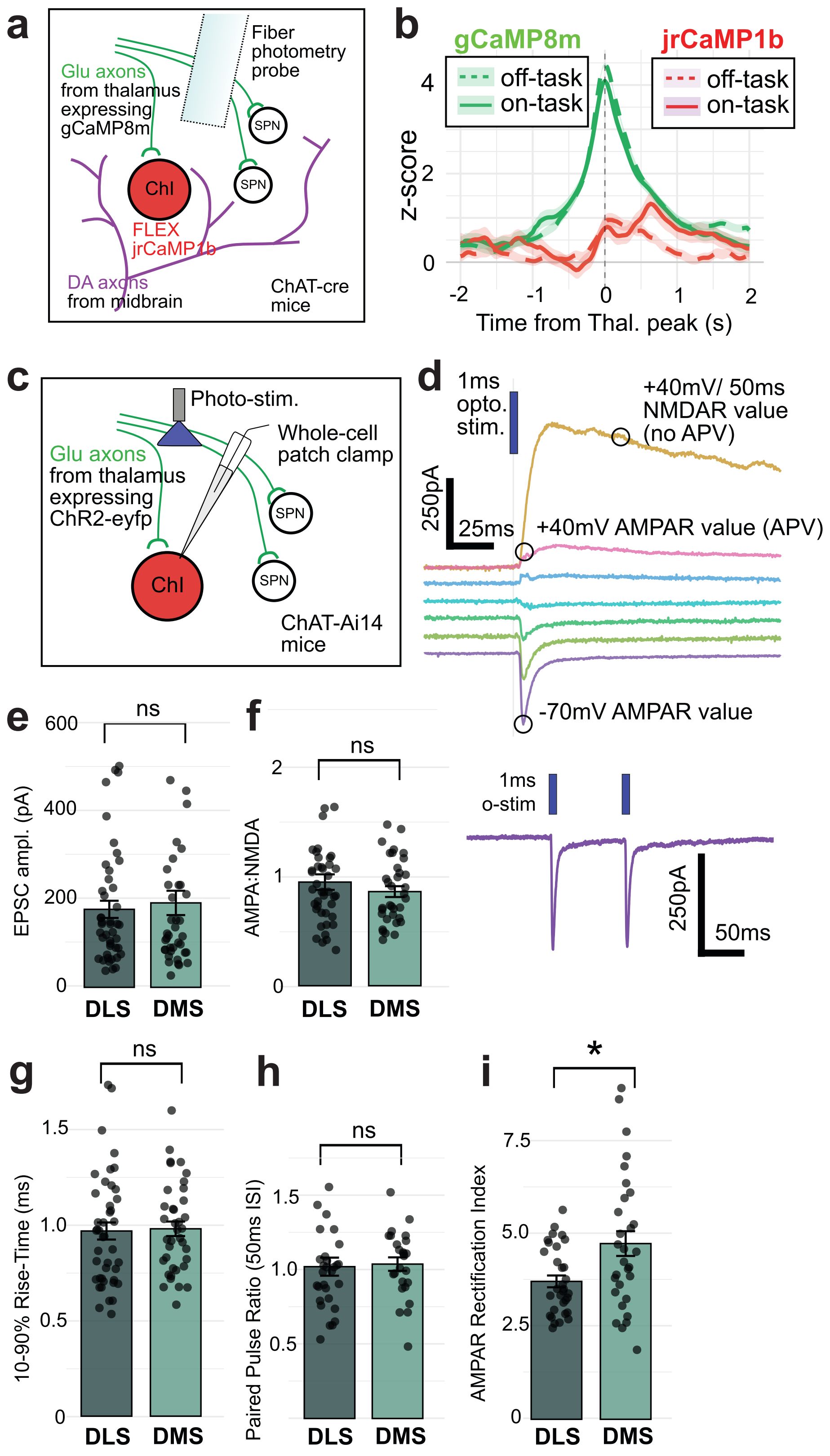

Supplementary Figure 6

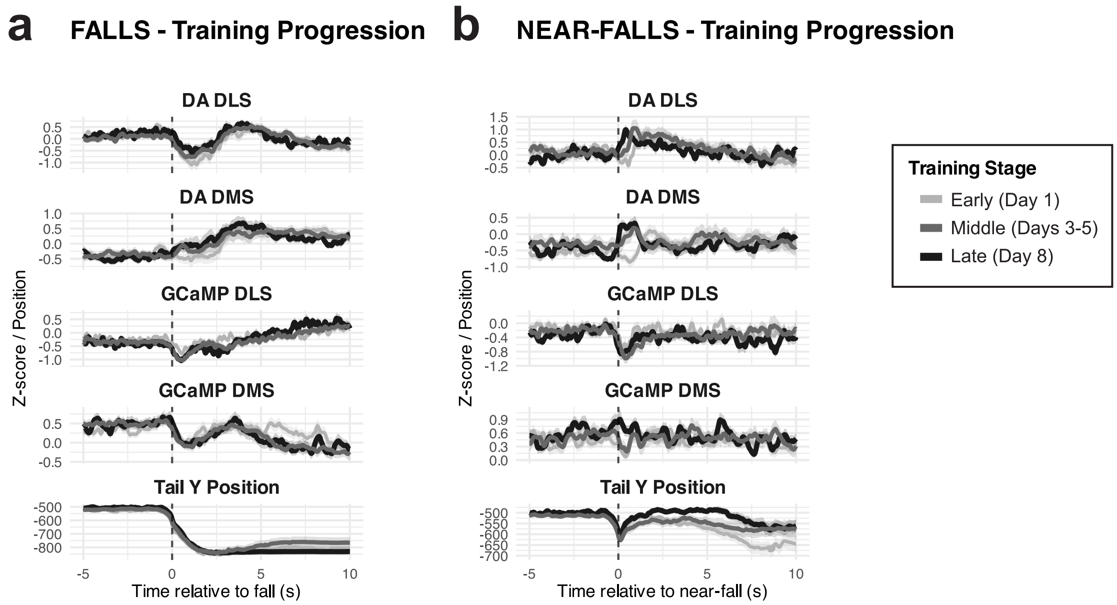

Supplementary Figure 7

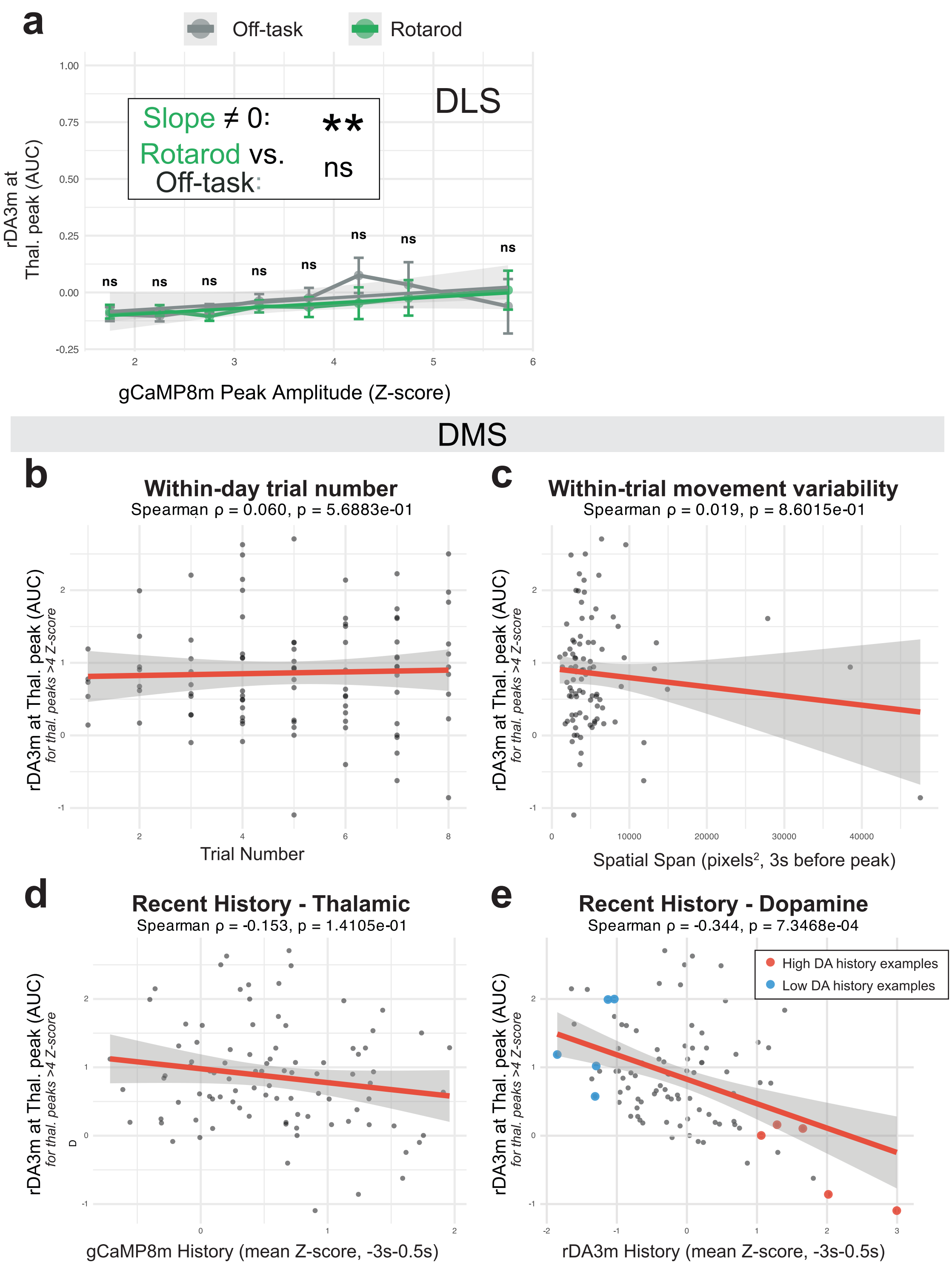

Supplement: Supplement 1 — Fig. S1: Anatomical and control information for photometry experiments in DMS and DLS a, DMS and DLS probe placements for all animals included in photometry experiments, snapped to nearest 250 μm plane. b, Example thalamic gCaMP8m injection showing rostrocaudal detail. Thalamic injections centered on the parafasicular (PF) nucleus, but also included rostral intralaminar central median (CM) and central lateral (CL) nuclei, which also project to striatum and were not considered separately in this study. c, Controls for dual-color photometry in DMS, signal processing, and data analysis pipeline. Black dashed line is rDA3m response to thalamic gCaMP8m peaks during blockade of rDA3m’s DA binding site with SCH-23390 (10mg/kg), as in Fig. 1e. Yellow dashed line is 560nm response to “peaks” in an animal where EYFP and mCherry were expressed in place of gCaMP8m and rDA3m. Orange dashed line is rDA3m response in real trial data, but using timestamps for gCaMP8m peaks which are arbitrarily shifted forward 4s; compare response against rDA3m response during genuine gCaMP8m peak timestamps in Fig. 1f. Fig. S2: Supplemental pharmacological data in DMS and DLS a, Thalamic axon gCaMP8m peak amplitudes before and after Mec. in DMS (paired Wilcoxon signed-rank test, p=1ns) and DLS (p=1ns) (n=5 mice). b, rDA3m peak amplitudes before and after Mec. in DMS (p=0.106ns) and DLS (p=0.787ns). c, rDA3m AUC during gCaMP8m peaks before and after MEC in DMS (p=0.106ns) and DLS (p=0.590ns). d, Direct comparison of average rDA response during gCaMP8m peaks pre- (purple) and post-Mec. (blue) in DMS, same data as in Fig 2b–c. e, Same as d, but in DLS. Fig. S3: Supplemental data for changes over days of rotarod training in DLS and DMS a, Average amplitude of thalamic axon gCaMP8m peaks in DLS during rotarod trials (green), off-task recordings (gray). Effect of training day on gCaMP8m peaks during rotarod trials (F(7, 35.03) = 3.54, p = 0.006**) and off-task recordings (F(7, 35.65) = 2.70, p = 0.024*) [file media-1.pdf]
