# Supplementary material for: Thalamus orchestrates local acetylcholine-dependent dopamine release in the learning striatum
Source: bioRxiv. 2026 May 8:2026.05.08.723861. Preprint. [Version 1] doi: 10.64898/2026.05.08.723861 (PMC13174615; doi:10.64898/2026.05.08.723861)

Supplementary Figure 1

a Photometry probe locations

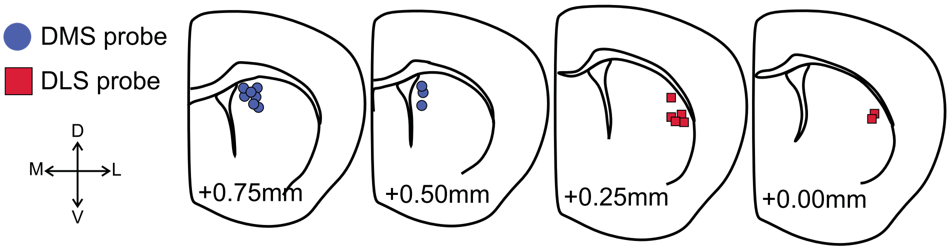

b Viral gCaMP8m expression in thalamus

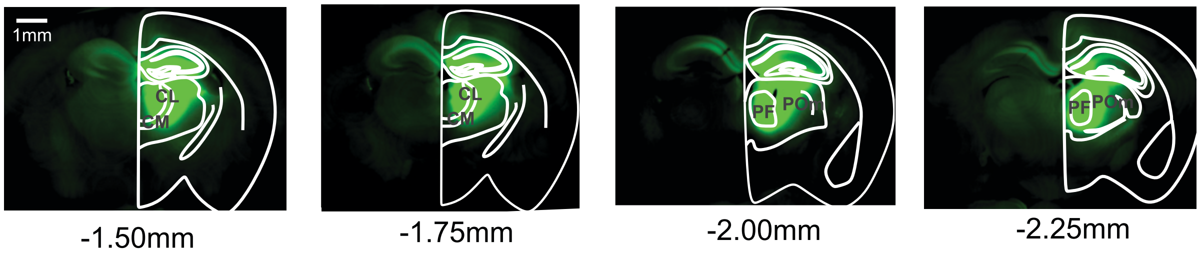

c Lack of DMS co-activation in controls

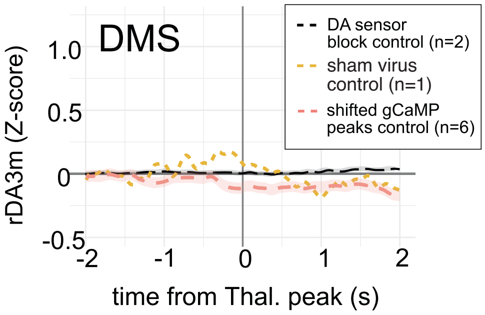

Supplementary Figure 2

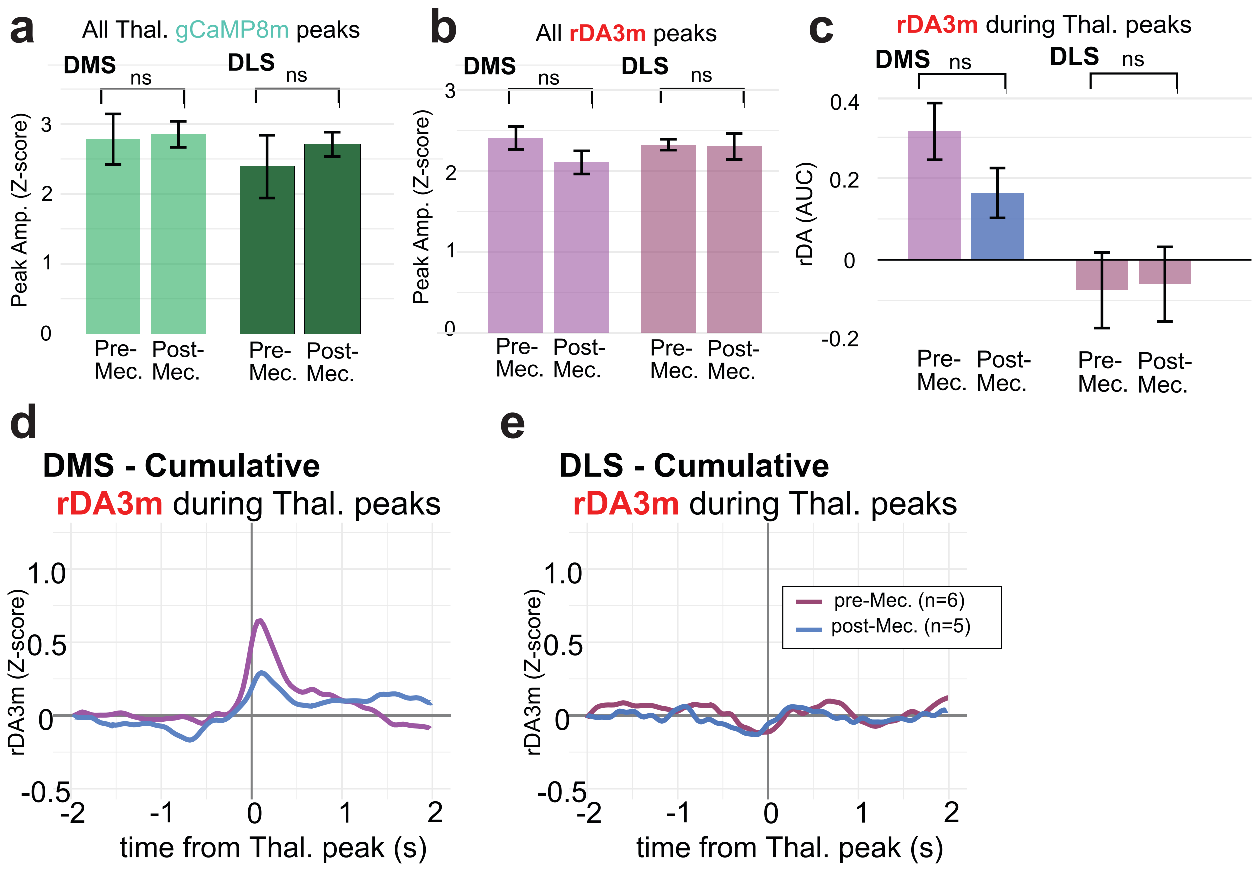

Supplementary Figure 3

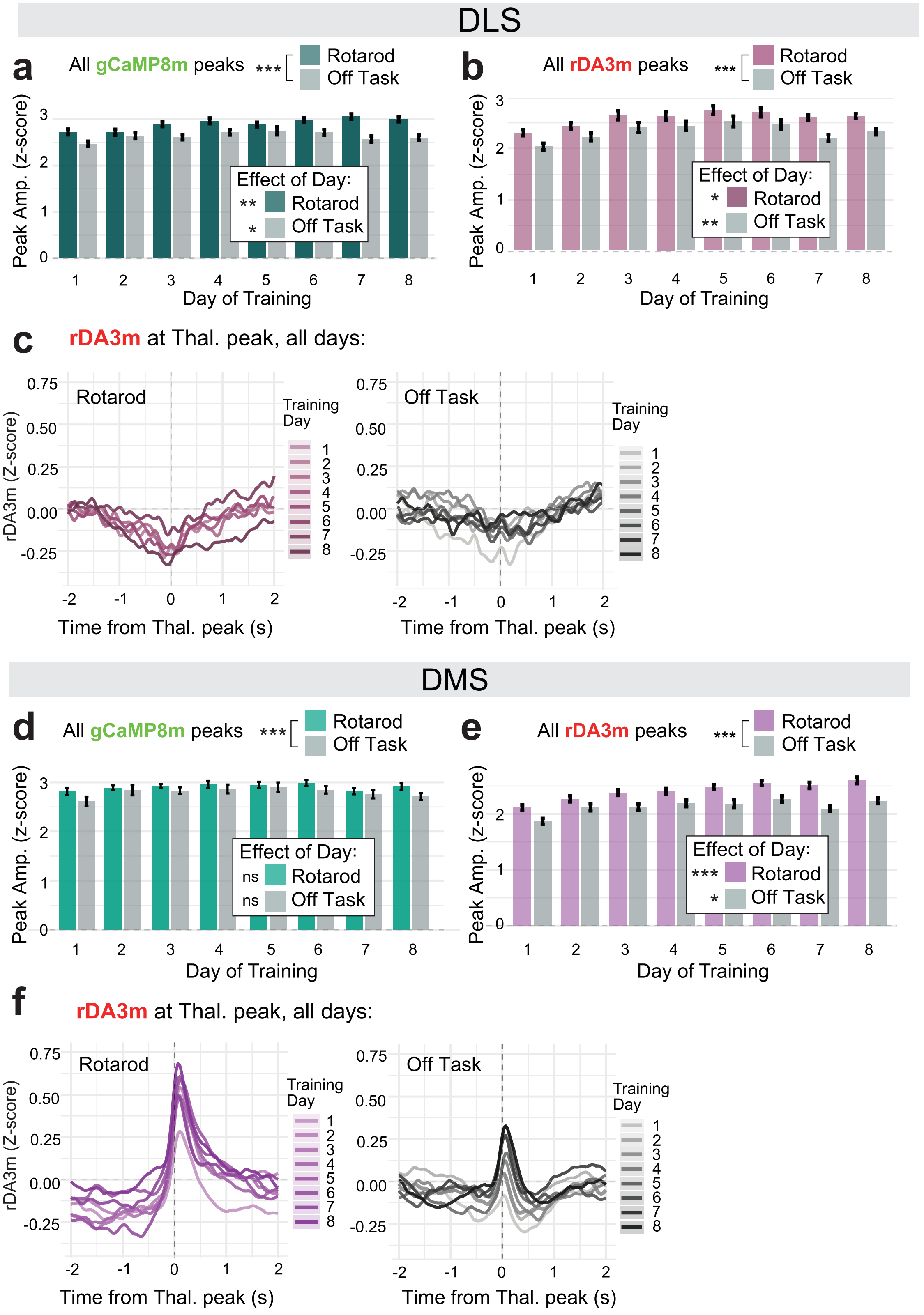

Supplementary Figure 4

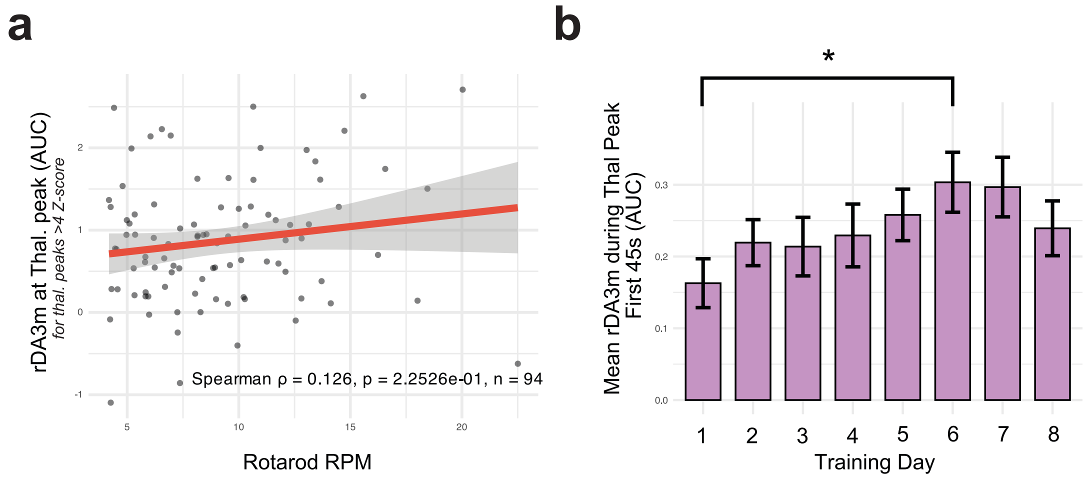

Supplementary Figure 5

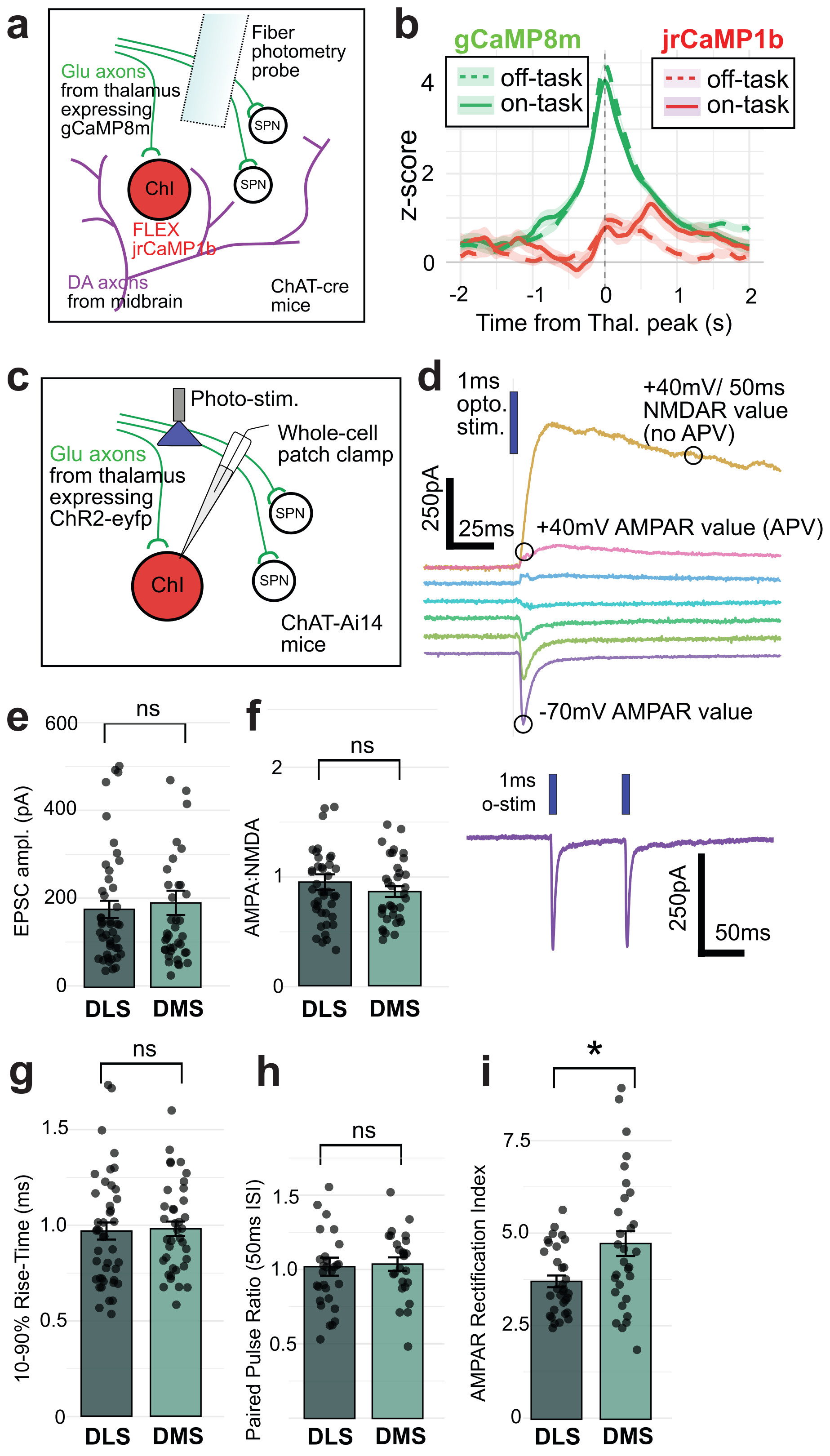

Supplementary Figure 6

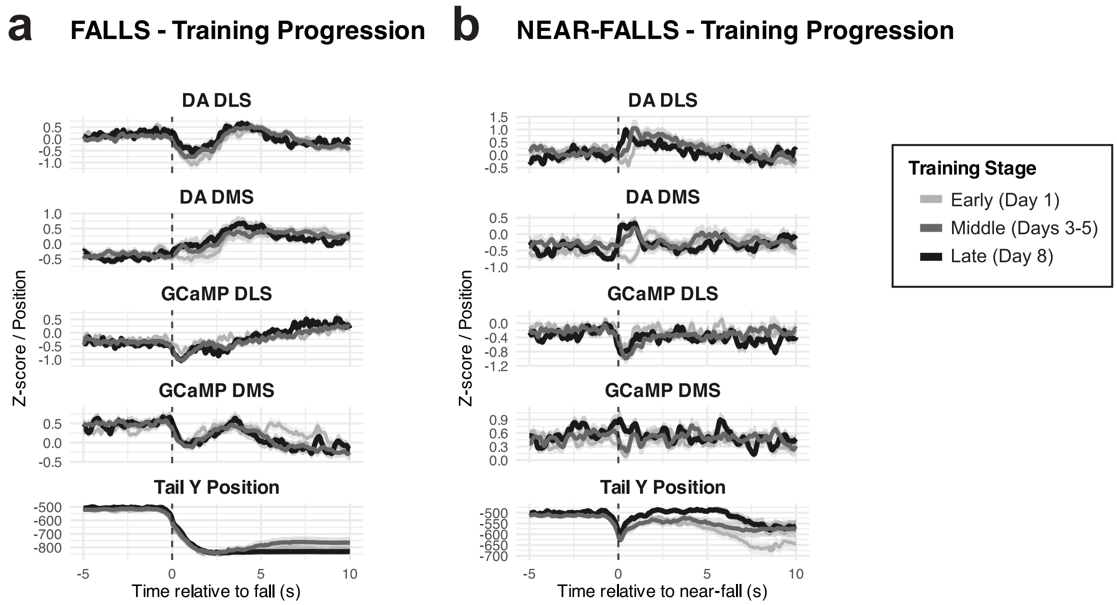

Supplementary Figure 7

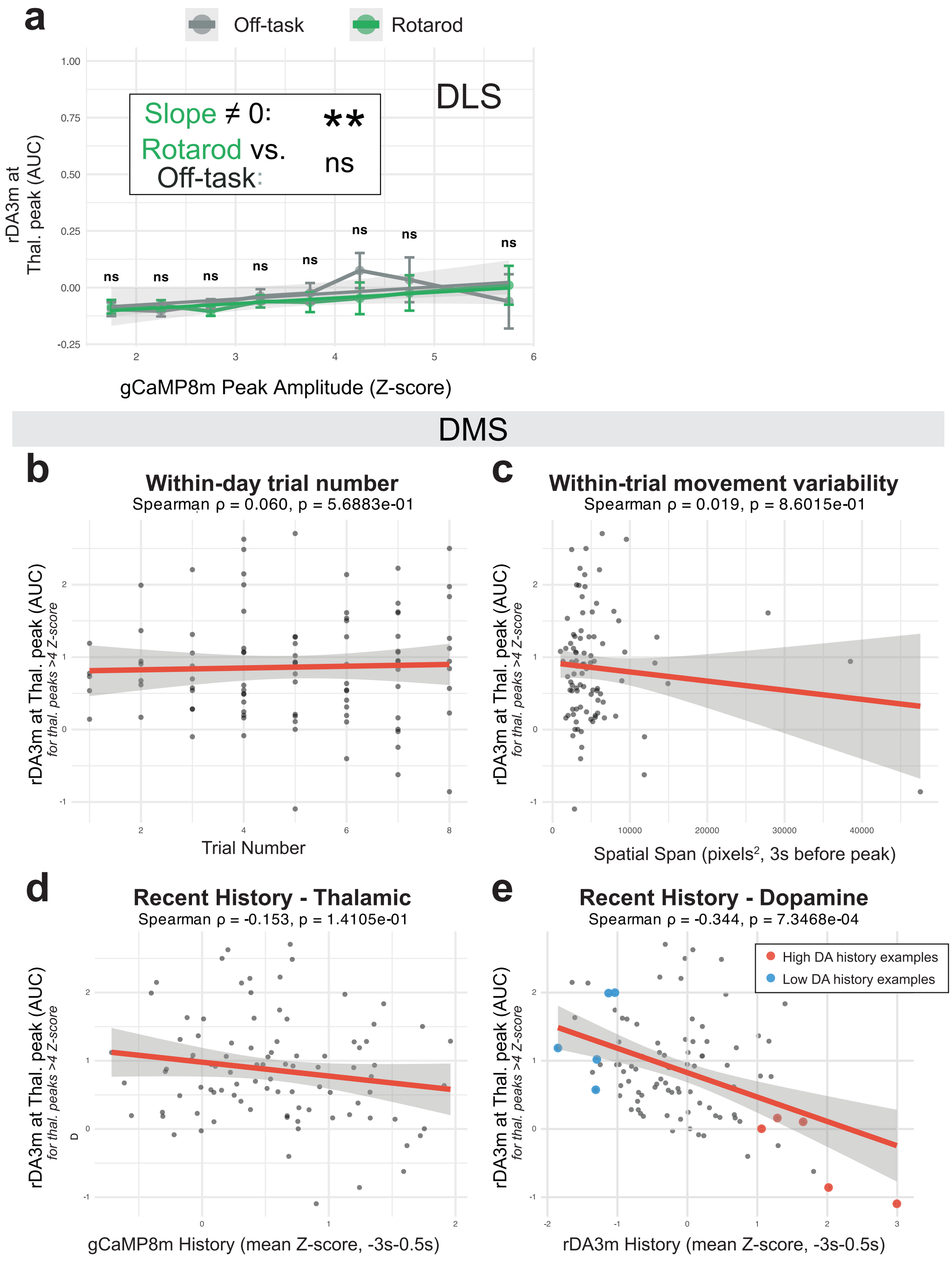

Supplement: Supplementary file 1 [file media-1.pdf]
